# Global brain analysis of minor hallucinations in Parkinson’s disease using EEG and MRI data

**DOI:** 10.3389/fnagi.2023.1189621

**Published:** 2024-01-17

**Authors:** Chuan Liu, Liangcheng Qu, Qixue Li, Yiting Cao, Jingping Shi, Chao Yu, Weiguo Liu, Kuiying Yin

**Affiliations:** ^1^Link Sense Laboratory, Nanjing Research Institute of Electronic Technology, Nanjing, China; ^2^Department of Neurology, Affiliated Brain Hospital of Nanjing Medical University, Nanjing, China

**Keywords:** minor hallucination, Parkinson’s disease, EEG, MRI, visual reconstruction

## Abstract

**Introduction:**

Visual hallucination is a prevalent psychiatric disorder characterized by the occurrence of false visual perceptions due to misinterpretation in the brain. Individuals with Parkinson’s disease often experience both minor and complex visual hallucinations. The underlying mechanism of complex visual hallucinations in Parkinson’s patients is commonly attributed to dysfunction in the visual pathway and attention network. However, there is limited research on the mechanism of minor hallucinations.

**Methods:**

To address this gap, we conducted an experiment involving 13 Parkinson’s patients with minor hallucinations, 13 Parkinson’s patients without hallucinations, and 13 healthy elderly individuals. We collected and analyzed EEG and MRI data. Furthermore, we utilized EEG data from abnormal brain regions to train a machine learning model to determine whether the abnormal EEG data were associated with minor hallucinations.

**Results:**

Our findings revealed that Parkinson’s patients with minor hallucinations exhibited excessive activation of cortical excitability, an imbalanced interaction between the attention network and the default network, and disruption in the connection between these networks. These findings is similar to the mechanism observed in complex visual hallucinations. The visual reconstruction of one patient experiencing hallucinations yields results that differ from those observed in subjects without such symptoms.

**Discussion:**

The visual reconstruction results demonstrated significant differences between Parkinson’s patients with hallucinations and healthy subjects. This suggests that visual reconstruction techniques may offer a means of evaluating hallucinations.

## 1 Introduction

Visual hallucination (VH) is a perceptual phenomenon characterized by the occurrence of false visual experiences while an individual is awake (Shine et al., 2014), resulting from erroneous neural coding in the brain. One of the common neurological diseases related to VH is Parkinson’s disease (PD) (Barnes and David, 2001; Diederich et al., 2009). In the past, complex VH has been roughly attributed to levodopa’s effects, thought to be a side effect, but as research progresses, it is now thought to be a symptom of a disconnect between the dorsal, ventral, and default-mode networks, mediated by the quality of visual input (Muller et al., 2014). As the disease progresses, CVH become more pronounced, evolving from simple visual disturbances such as flashes or geometric patterns to vivid depictions of colorful animals and figures. This progression can lead to a gradual loss of the ability to differentiate between CVH and reality (Fénelon et al., 2000). However, research has indicated that even in the early stages of PD, prior to medication treatment, some patients may exhibit mild VH symptoms, which are referred to as Minor Hallucinations (MH). Minor hallucinations include three aspects: existential hallucinations, through sexual hallucinations, illusions (Lenka et al., 2019).

In order to analyze the data and reconstruct the vision, it is necessary to understand the mechanism of visual hallucination. It is a good way to study CVH from a model-building point of view. For example, Collerton et al. (2023) provide eight models to explain the mechanism of CVH. With the development of research, the current mainstream consensus on the mechanism of Parkinson’s disease (PD). CVH is the theory that bottom-up and top-down information flow transmission is impaired, bottom-up: impaired transmission of visual information through the optic nerve-optic chiasm-lateral geniculate body-primary visual cortex; Top-down: impaired transmission of visual information through the ventral and dorsal advanced processing pathways of visual information; At present, we prefer to study the processing of advanced information from the aspect of brain network. Recruiting patients is a difficult task, and as hallucinations become more severe, information gathering becomes more difficult. We try to recruit a certain number of patients with minor hallucinations. A study investigating structural and functional changes associated with MH found that disruption of the internal organization of the DMN and its precise balance with the attentional control network is responsible for minor hallucinations in patients with Parkinson’s disease (Bejr-kasem et al., 2019). This is consistent with the attention network hypothesis for CVH proposed by Shine et al. (2015) and Caviness (2014). It is concluded that MH and CVH have similar structural and functional correlations. It is reliable to study the mechanism of visual hallucination in patients with minor hallucinations.

In order to study the mechanism of MH subjects with PD, a total of 13 PD patients with MH, 13 PD patients without hallucinations, and 13 healthy individuals were recruited for the present study. EEG and MRI data were collected and analyzed across all brain regions to investigate the neural correlates of MH in PD. The aberrant brain regions were found to contain neural coding information related to the experience of hallucinations. Furthermore, visual reconstruction techniques were employed to validate the association between MH and EEG data derived from these abnormal brain regions.

## 2 Materials and methods

### 2.1 Participants and stimuli

We recruited 13 PD patients with minor hallucinations (PD-MH), 13 PD Patients without hallucinations (PD), and 13 healthy controls (HC), respectively. PD patients were grouped into hallucinations and non-hallucinations according to the Unified Parkinson’s Disease Rating Scale (UPDRS), Part I, Item 2(Goetz et al., 2003). The participants who got score of 2 or more were grouped into hallucinations, the others were grouped into non-hallucinations. All subjects were recruited at Nanjing Medical University Affiliated Brain Hospital and all experimental procedures were approved by the Ethics Committee of Affiliated Brain Hospital of Nanjing Medical University (Ethics approval number: 2019-KY018-01). The inclusion criteria is Mini-Mental State Examination (MMSE) score of more than 17. Exclusion criteria included patients with eye diseases; limitations in MRI scanning, such as claustrophobia or pacemaker implantation; a history of cerebral infarction or brain tumor.

The demographic and clinical characteristics of all participants are presented in Table 1. There were no significant differences in age, gender, education level, cognitive ability, disease duration, and H-Y staging between the PD-MH group and the PD group. It is important to note that all PD patients had not previously received levodopa treatment, thus ruling out any potential drug effects.

**Table 1:** Demographic and clinical data of subjects.

Group	Number	Age	Disease duration (years)	Education level (years)	MMSE	MOCA	Gender	Hoehn & Yahr Stage	Characteristics of MH
HC	1	52		9	29	27	Female		
HC	2	61		10	29	25	Female		
HC	3	53		6	28	17	Male		
HC	4	50		6	29	22	Female		
HC	5	62		9	28	23	Female		
HC	6	51		9	30	27	Male		
HC	7	65		9	29	25	Male		
HC	8	52		9	30	23	Female		
HC	9	55		9	29	25	Female		
HC	10	62		12	29	27	Female		
HC	11	62		0	26	16	Male		
HC	12	63		9	29	25	Female		
HC	13	52		9	30	30	Male		
PD	14	70	0	9	28	22	Female	2	
PD	15	51	0	6	26	20	Male	1	
PD	16	53	1	16	28	29	Female	1	
PD	17	53	2	6	28	17	Male	1	
PD	18	55	0.5	9	25	19	Female	2.5	
PD	19	55	5	0	23	13	Female	2	
PD	20	71	3	9	29	26	Female	1.5	
PD	21	57	1	9	30	26	Male	2	
PD	22	59	1	0	22	13	Female	2.5	
PD	23	63	1	9	26	20	Male	1	
PD	24	56	0.5	5	30	24	Male	1.5	
PD	25	61	5	12	30	28	Female	1	
PD	26	63	0.5	13	29	27	Female	1	
PD-MH	27	65	2	0	21	15	Female	2	Visual illusions
PD-MH	28	56	2	12	27	16	Male	2	Visual illusions
PD-MH	29	66	2	9	27	23	Male	1	Unknown
PD-MH	30	56	4	5	29	22	Female	2	Unknown
PD-MH	31	61	1	8	29	18	Female	1.5	Unknown
PD-MH	32	74	3	8	27	19	Male	1	Visual illusions
PD-MH	33	66	2	11	26	23	Female	1.5	Unknown
PD-MH	34	67	2	13	29	25	Male	1	Unknown
PD-MH	35	55	3	5	29	18	Female	1.5	Passage hallucinations
PD-MH	36	65	3	9	28	21	Female	1.5	Unknown
PD-MH	37	50	1	12	25	16	Female	2.5	Passage hallucinations
PD-MH	38	66	3	0	19	13	Female	1.5	Visual illusions
PD-MH	39	66	3	0	22	13	Female	2.5	Presence hallucinations

In this study, both resting-state and task-state data were collected from all participants. The resting-state data acquisition lasted for a duration of 60 s with eyes closed. For the task-state data collection, participants were instructed to sit in a room where objects and people were arranged in a complex manner. The task required participants to verbally describe the objects and people they observed in order to assess their visual orientation and content. During the task, a camera was positioned directly behind the participants’ eyes, capturing the visual scenes they were observing in real-time. In terms of MRI data collection, a sequence of resting-state data was obtained. During the MRI session, participants were in a resting state, characterized by a clear and relaxed state of mind, with unknown eye closure. No task-state magnetic resonance data was collected, and no specific gaze point was recorded.

### 2.2 Data collection and processing

The EEG recording was taken with the 64-channel EGI GES systems using AgCl electrode (10–10 standard mapping). The data was sampled at a frequency of 1,024 Hz, and filtered between 0.1 and 50 Hz by FIR filter. Noisy or unusable channels was removed according to the Nina rule (Bigdely-Shamlo et al., 2015). The data was processed using eeglab toolkit in MatLab (MathWorks). Independent Component Analysis (ICA) was used to separate the noise components and the data source components. Then the bad components of EEG data are manually removed, and finally restore the sequence EEG data.

MRI was obtained using a Siemens 3.0 T singer scanner (Siemens, Verio, Germany) and an 8-channel radio frequency coil at the Nanjing Medical University Affiliated Brain Hospital. The 3D-SPGR sequence was used to obtain three-dimensional T1-weighted images in the sagittal plane with the following parameters: TE = 3.34 ms; TR = 2,530 ms; flip angle = 7°; 128 sagittal surface slices; 1.33 mm slice thickness; matrix = 256 × 256. Functional images were collected using T2-weighted single-shot EPI sequences: 240 time points; TE = 30 ms; TR = 2000 ms; FOV = 240 × 240 mm 2; matrix = 64 × 64; flip angle = 90°; 30 axes 3.0 mm thick; section gap = 0 mm. The MRI data adopts the resting-state fMRI data processing assistant toolkit DPARSF based on the statistical parameter mapping software SPM8 to be processed. To ensure data quality, the first 10 moments of each subject’s data were discarded. We performed slicing timing and motion calibration on the rest of the images. Based on head motion recordings, all participants had a maximum displacement of less than 2.0 mm on the x, y, or z axis, and a maximum angular rotation of less than 2° on each axis. After spatial normalization of the T1 space, all images were resampled to 3 × 3 × 3 mm-sized voxels and spatially smoothed using a Gaussian filter with a half maximum width of 4 mm. The fMRI data were then subjected to temporal bandpass filtering (0.01–0.08 Hz) to remove low-frequency drift and physiological high-frequency noise.

### 2.3 EEG and MRI data analysis

The Fast Fourier Transform (FFT) was utilized to analyze the frequency distribution of EEG data during the task-state. The power ratio for each channel was calculated across different frequency bands, including delta (1–3 Hz), theta (4–7 Hz), alpha (8–12 Hz), beta (12–30 Hz), and gamma (>30 Hz) (Ramadan and Vasilakos, 2017). To account for impedance differences between channels, a single medium channel was used to adjust the bias. The threshold for each channel was determined as 1.5 times the mean difference within each group of subjects. For instance, when analyzing the delta power ratio in the frontal area electrodes of the three groups (PD-MH, PD, HC), three average values $x_{1},x_{2},x_{3}$ were obtained, and the threshold for PD-MH relative to PD in the frontal lobe with differential channels was calculated as 1.5 * ( $x_{1}$ - $x_{2}$ ).

In the processing of MRI data, the ICA method was employed using functional magnetic resonance imaging (fMRI) data. Seven brain functional sub-networks were identified based on the AAL template for intra-network comparison. The Shapiro–Wilk test was used to assess the normality of the data, and the ANOVA test was conducted to analyze the significance of the data across the three groups. Multiple comparisons were then performed to examine the differences between groups. A significance level of *p* < 0.05 was considered statistically significant.

Additionally, indices of fractional anisotropy (FA) and mean diffusivity (MD) were calculated, and whole-brain fiber tract tracking was conducted using diffusion tensor imaging (DTI) based on the MRI data (Le Bihan et al., 2001; Alexander et al., 2007; Thomason and Thompson, 2011). This analysis was performed on the DAN and VAN networks. The fiber bundle orientation map of specific brain regions was determined using the ICBM-DTI-81 template provided by Johns Hopkins University.

### 2.4 Visual reconstruction analysis based on EEG signal

**Figure 1:**
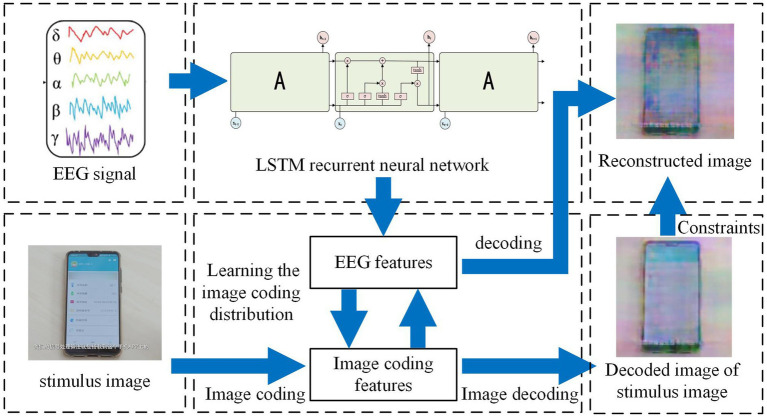
Visual reconstruction algorithm flow chart.

A deep learning network is capable of computing features of input information through multiple iterative processes. In the context of vision-based reconstruction technology, deep network technology is employed to simultaneously calculate two sets of deep network structures in parallel. One network is responsible for computing features of the input EEG signals, while the other network supervises the original stimuli. The iterative EEG features are then transmitted to the feature decoder, resulting in the output of a reconstructed visual stimulus image (Qu et al., 2021). The algorithm network flow chart depicting this process is illustrated in Figure 1.

In our study, visual stimuli in the form of pictures were utilized for our subjects. We collected EEG data for a duration of 500 ms following the presentation of the stimuli. Subsequently, the EEG signal underwent offline preprocessing as described earlier. The preprocessed signal was then fed into the first group of Long Short-Term Memory (LSTM) recurrent neural networks to extract the features present in the EEG data. Simultaneously, the stimulation image associated with the EEG signal was inputted into the second group of neural network VGG, which performed calculations for encoding and decoding models of the stimuli images, thereby extracting the features of the image coding process. These features were then transmitted to the first group of LSTM networks, resulting in the acquisition of EEG features related to the stimulus image features. At this stage, the EEG features associated with the stimulus image features were sent to the constructed VGG model, where image decoding and reconstruction were carried out, ultimately producing the reconstructed image.

**Figure 2:**
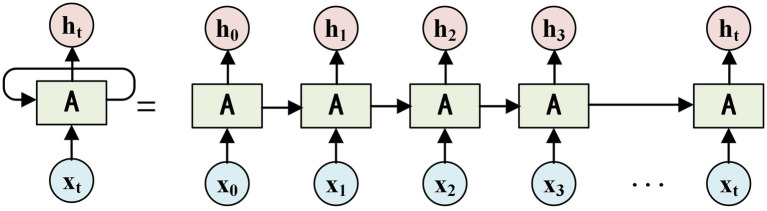
LSTM network model.

LSTM is a recurrent neural network (RNN) that incorporates hidden computational gates. RNNs are designed to handle data with semantic associations, such as text sentences, semantics, reasoning, and stocks, which are logically related. EEG signals, in particular, are suitable for recurrent neural networks as they contain human physiological behaviors and logical patterns. However, when dealing with large amounts of data, the iterative calculation of RNNs may result in gradient explosion. To address this issue, a special type of RNN called LSTM, which includes hidden gate settings, is utilized. The network structure model of LSTM is depicted in Figure 2.

In Figure 2, a hidden gate structure called the tan gate is added to the input data h. This hidden gate structure performs long-short-term memory sequence calculations each time the input data h is processed. During each calculation, a parameter related to the forward calculation is randomly omitted, and the input parameter is repeated twice. The resulting tan calculation is then fed into the next hidden gate h. By iteratively calculating the LSTM recurrent neural network, the features of the EEG signals fed into the neural network can be outputted as $f_{E}$ .

Simultaneously, the stimulation image associated with the EEG signal is sent to a second neural network called VGG. This network is responsible for encoding and decoding models of stimuli images, extracting features from the image coding process. VGG is a deep encoding and decoding computing network and is considered a classic representative network structure of convolutional neural networks (CNNs). It possesses a deep network structure and can perform numerous iterative calculations on input images through operations such as convolution, pooling, and activation. This allows VGG to compute the underlying features of the input image and construct an encoding and decoding model for similar images. The deeper network structure of VGG enables it to encode and compress images to a higher degree. However, one drawback is that the reconstructed images after decoding may exhibit lower accuracy.

The encoding and decoding model of the input images was constructed using the VGG model. The forward structure of the network was then utilized to generate the encoding results of the stimulus image corresponding to the input’s EEH time series. The results $f_{P}$ were sent to the first group of network LSTM as the time-stamp for image encoding results and for calculating the supervision loss of the EEG feature extraction network. The supervision mode employed the Archirid loss Formula (1).


(1) $$\frac{1}{2N}{\sum_{i=1}^{N}\left\|x_{i}^{1}-x_{i}^{2}\right\|}_{2}^{2}$$

After the supervision of Equation (1) and the stimulus image, the EEG signal sent to the LSTM network would eventually output EEG features $f_{E}$ related to the stimulus image feature.

At this time, the EEG features $f_{E}$ associated with the stimulus image features were sent to the constructed VGG model, and the decoding calculation of this model was regarded as image features, $f_{E}$ and image decoding and reconstruction were performed on them.

The original image was used as supervision, and MES loss was applied to the reconstructed image as decoding supervision, as shown in Formula (2):


(2) $$E\left(w\right)=\frac{1}{2}\overset{N}{\underset{n=1}{\sum }}{\left\{y\left(x_{n},w\right)-t_{n}\right\}}^{2}$$

Then, the decoded image with original image supervision was used as the output of the decoder, that is, the reconstructed image is obtained.

For the purpose of our study, we collected EEG data spanning 500 ms from the PD-MH subject during approximately 1,200 experiments, which encompassed instances where the partner either faced the subject or did not face the subject. Out of these experiments, 1,000 were used as a training set and 200 were allocated as a test set.

The structural similarity index (SSIM) was employed to assess the accuracy of the reconstruction as shown in Formula (3).


(3) $$\mathrm{SSIM}\left(x,y\right)=\frac{\left(2\mu_{x}\mu_{y}+c_{1}\right)\;\left(2\sigma_{xy}+c_{2}\right)}{\left(\mu_{x}^{2}+\mu_{y}^{2}+c_{1}\right)\;\left(\sigma_{x}^{2}+\sigma_{y}^{2}+c_{2}\right)}$$

where, μ*_x_* is the average value of x, $\sigma_{x}^{2}$ is the variance of x, μ*_y_* is the average value of y, $\sigma_{y}^{2}$ is the variance of y, $\sigma_{xy}$ is the covariance of x and y, $c_{1},c_{2}$ are constants.

## 3 Results

### 3.1 EEG analysis results

**Figure 3:**
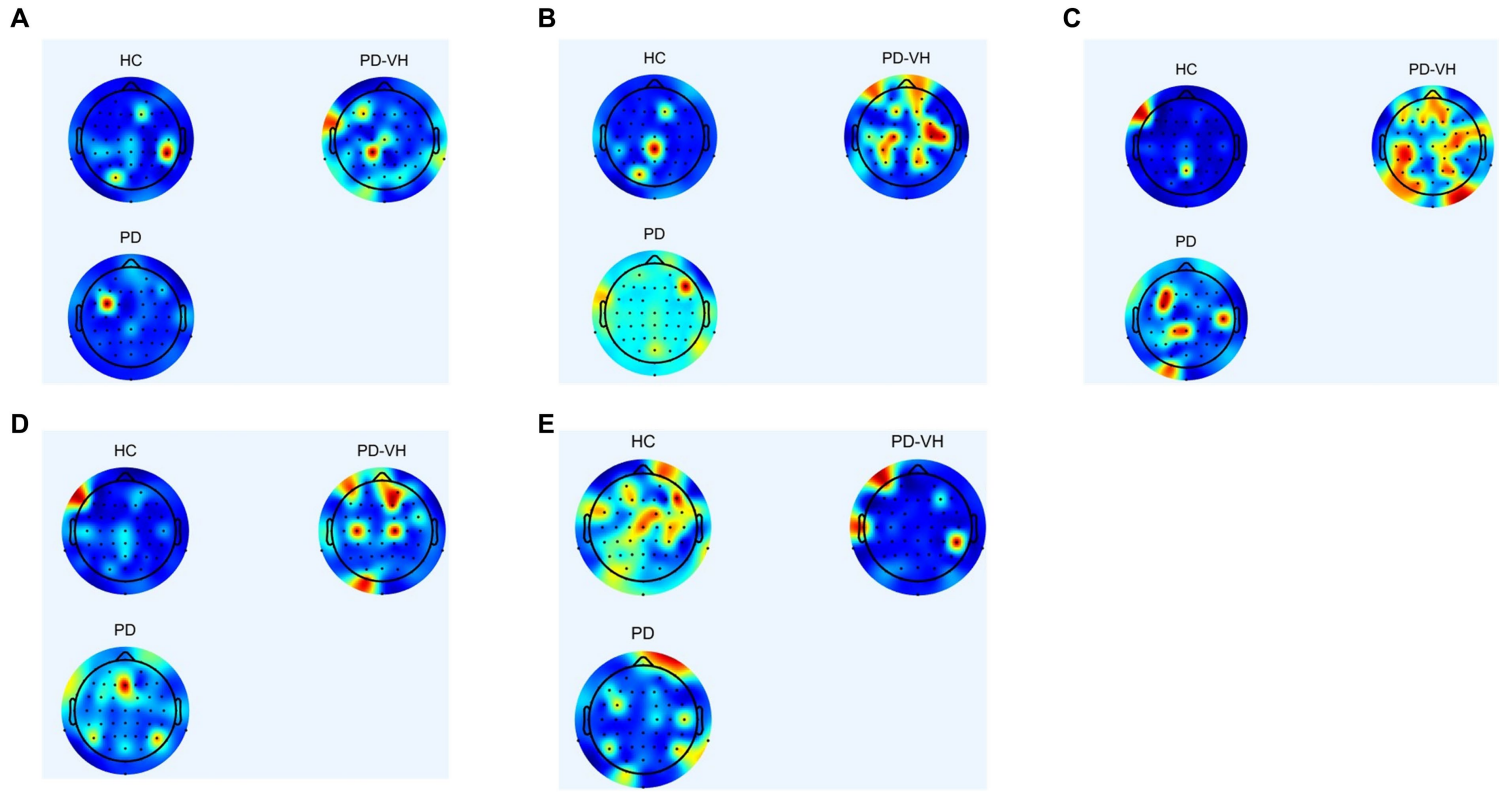
**(A)** δ frequency band **(B)** θ frequency band **(C)** α frequency band **(D)** β frequency band **(E)** γ frequency band.

Figure 3 depicts the EEG mapping results for 13 patients with PD-MH, 13 patients with PD, and 13 HCs across various frequency ranges. The color scheme in the figure represents the average intensity, with red indicating high intensity and blue indicating low intensity.

In comparison to the healthy controls, the PD-MH patients exhibited an elevated power ratio in the α and θ frequency bands, particularly at the junction of the frontal lobe, occipital lobe, fronto-parietal junction, and occipital-parietal junction. Conversely, a decrease in power was observed in the γ frequency band, specifically in the frontal lobe, parietal lobe, and occipital lobes.

### 3.2 MRI analysis results

Table 2 presents the results of a multiple-comparison test conducted on three groups of subjects in the analysis of MRI functional network. The table records the connections between brain regions, denoted as Node-a and Node-b. The term “excellent” indicates that the functional connectivity of the PD-MH group has a significant advantage, while the absence of this term suggests a significant disadvantage in functional connectivity for the PD-MH group.

**Table 2:** Results of significant differences in functional networks.

	PD-MH:HC		PD-MH:PD	
	Node-a	Node-b	Frontal_Mid_L	Frontal_Inf_Tri_R
Enhanced	Frontal_Sup_L Frontal_Sup_R Frontal_Mid_L	Calcarine_L Cuneus_L Calcarine_R Occipital_Inf_R Calcarine_R	Frontal_Inf_Tri_R	Supp_Motor_Area_L Postcentral_L Heschl_L Calcarine_L Cuneus_L Occipital_Mid_R Occipital_Inf_R Temporal_Sup_L Temporal_Pole_Sup_L
	Frontal_Mid_R	Supp_Motor_Area_R Postcentral_L, Postcentral_R Temporal_Mid_R Occipital_Inf_R Calcarine_L, Calcarine_R Putamen_L Amygdala_R	Cingulum_Ant_R	Temporal_Pole_Sup_L
	Frontal_Sup_Medial_R	Olfactory_R Occipital_Inf_R Angular_R	Occipital_Inf_R	Frontal_Inf_Oper_R Frontal_Inf_Tri_L Frontal_Inf_Tri_R
	Occipital_Inf_R	Frontal_Inf_Oper_L Supp_Motor_Area_L, Supp_Motor_Area_R Insula_R SupraMarginal_R Fusiform_R Hippocampus_L, Hippocampus_R Lingual_L, Lingual_R Caudate_R Putamen_L, Putamen_R Pallidum_L, Pallidum_R Thalamus_R Cingulum_Ant_L, Cingulum_Ant_R Cingulum_Mid_R Cingulum_Post_L		
Attenuated	Temporal_Pole_Mid_L	Temporal_Pole_Sup_R Amygdala_L	Temporal_Pole_Mid_L	Hippocampus_L Hippocampus_R ParaHippocampal_L Amygdala_L Temporal_Sup_R Temporal_Pole_Sup_R
			Frontal_Sup_Orb_R	Occipital_Sup_R Occipital_Mid_R

The PD-MH group exhibited significantly enhanced connectivity between the frontal gyrus and middle frontal gyrus compared to the HC group. These connections also involved the Calcarine, and enhancements were observed in the parietal and occipital lobes. In comparison to the PD group, the PD-MH group showed enhancements in a limited number of brain regions, such as Occipital gyrus.

Table 2 displays complex network nodes that require further investigation to establish their functional connections. To this end, seven functional networks were selected for significance analysis, and the results are presented in Table 3. The second and third columns of Table 3 indicate the *p*-values associated with the significance of the functional networks.

**Table 3:** Comparison results of functional network sub-connections of the three groups of subjects.

	PD-MH:HC	PD-MH:PD
Sensorimotor system	0.076	0.105
Central executive network Central executive network	0.547	0.621
DMN	0.008	0.025
Highlight the network Salience network	0.681	0.775
DAN _	0.017	0.032
VAN _	0.011	0.028
Limbic/paralimbic system	0.057	0.348

Table 3 reveals substantial differences among the DMN, DAN, and VAN networks for the three groups of subjects, prompting a specific investigation into these networks.

In the analysis of the DMN, the functional connections of the posterior cingulum, medial prefrontal cortex, angular gyrus center dorsal medial subsystem, and medial temporal subsystem were compared among the three groups of subjects. The results are displayed in Table 4. It was observed that the DMN network in the PD-MH group exhibited significant enhancement, particularly in the dorsal medial subunit centered on the medial prefrontal cortex.

**Table 4:** Comparison of results of DMN network connection among three groups of subjects.

PD-MH: HC		PD-MH: PD	
Node - a	Node - b	Node - a	Node - b
Cingulum_Post	Frontal_Sup_Medial Temporal_Sup Temporal_Mid_R Temporal_Inf_R	Cingulum_Post	Frontal_Sup_Medial Temporal_Sup_L _ _ Temporal_Pole_Sup_L
Frontal_Sup_Medial	Angular Temporal_Sup Temporal_Mid_R Temporal_Inf_R Temporal_Pole_Sup	Frontal_Sup_Medial	Angular Temporal_Sup Temporal_Mid Temporal_Inf Temporal_Pole_Sup
Angular_L	Temporal_Mid_L Temporal_Inf_L Temporal_Pole_Sup_L	Angular_L	Temporal_Sup Temporal_Mid_L Temporal_Pole_Sup
Cingulum_Post	Parietal_Inf_L	Cingulum_Post	Parietal_Inf_L
Frontal_Sup_Medial	Hippocampus ParaHippocampus Parietal_Inf	Frontal_Sup_Medial	Hippocampus ParaHippocampus Parietal_Inf

**Figure 4:**
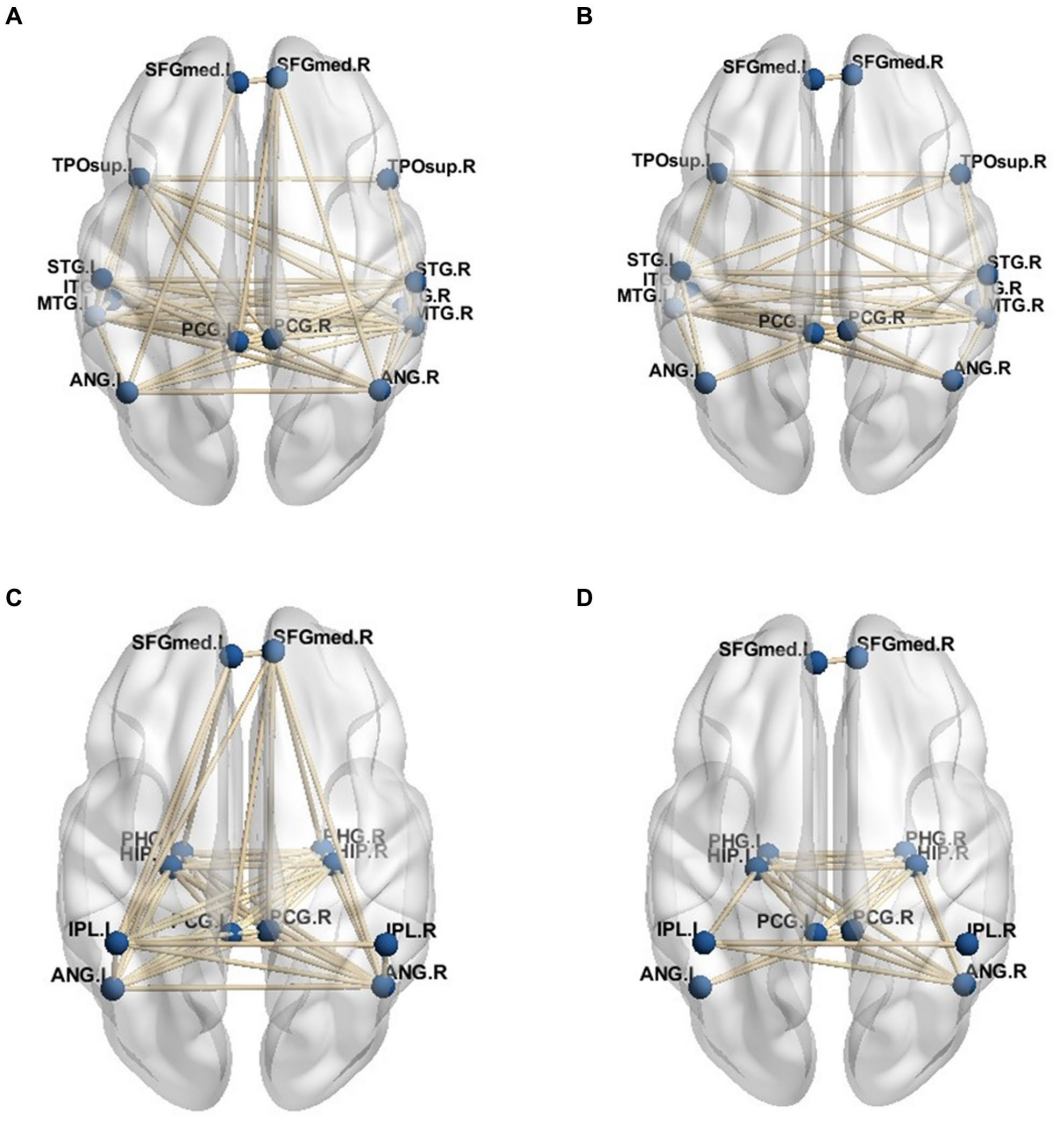
**(A)** dorsal medial subsystem network of PD-MH group **(B)** dorsal medial subsystem network of HC group **(C)** temporal -medial subsystem network of PD-MH group **(D)** temporal -medial subsystem network of HC group.

Given the connection strength is close between the PD-MH group and the HC and PD groups, a connection strength above 0.8 in functional connectivity was considered an effective threshold. Consequently, the average DMN of the PD-MH group with hallucinations and the HC group was determined. The network connection traces are depicted in Figures 4A–D. Figures 4A,B represent the network diagrams of the dorsal-medial subsystems, while Figures 4C,D represent the temporal-medial subsystems. The default network of PD-MH patients exhibited greater connection complexity than that of the HC group, particularly in the dorsal subsystem.

**Figure 5:**
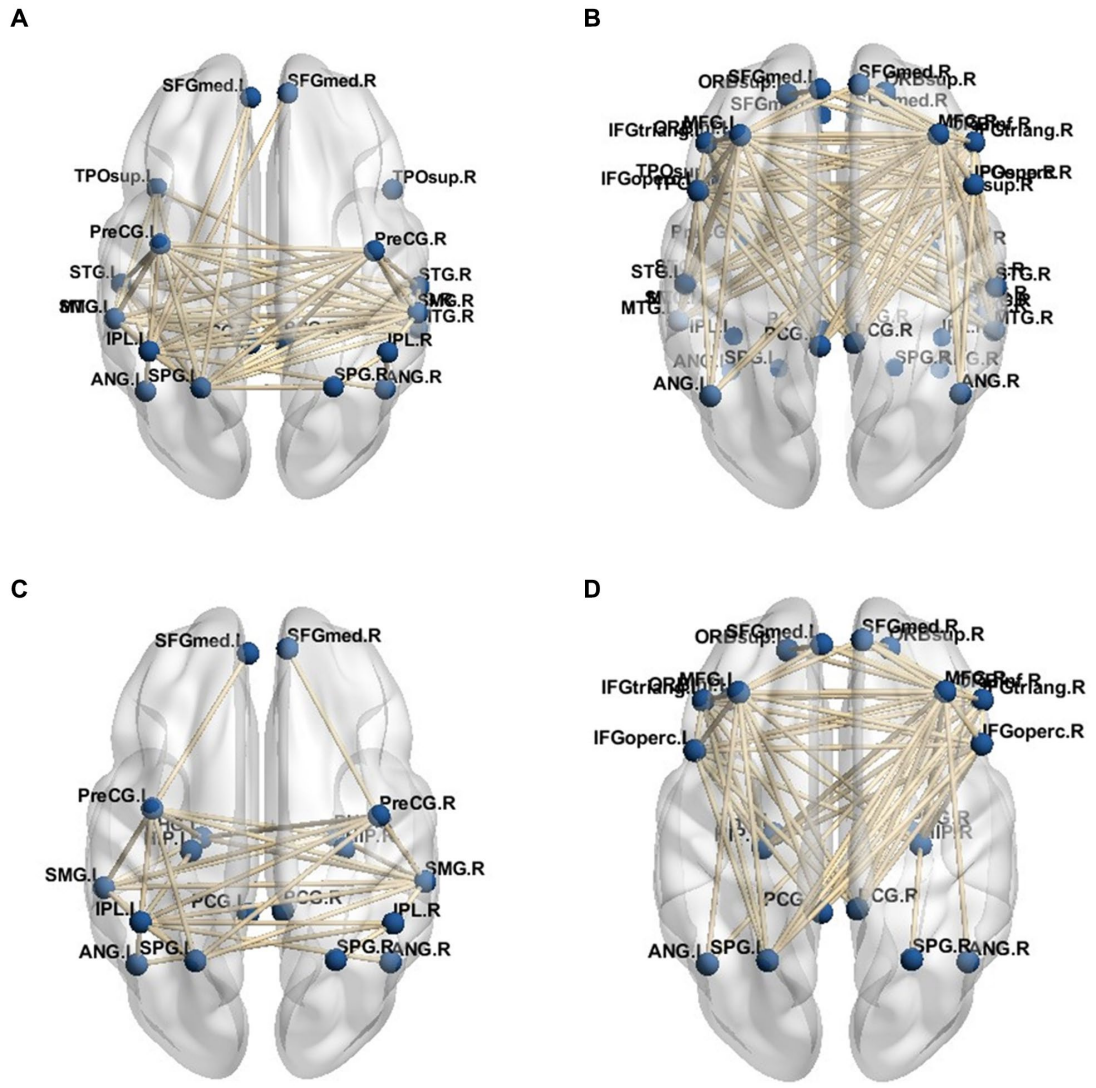
DMN subsystem network connection. **(A)** Dorsal attention network and dorsal medial subsystem. **(B)** Ventral attention network and dorsomedial subsystem. **(C)** Dorsal attentional network and temporal - medial subsystem. **(D)** Ventral attention network and temporal - medial subsystem.

For the functional sub-networks, DAN, and VAN of visual information processing, the average connection result with DMN also used 0.8 as the threshold, and the network connection is depicted in Figures 5A–D. This figure only includes the connection between the visual information processing sub-network and the DMN network, not includes the connection of the DMN network itself. As can be seen from the figure, the VAN network was more closely connected with the DMN network than the DAN network, while in the connection with the DMN network, the dorsal-medial system was more closely connected with the attention network, namely PD - MH In the network connection of group patients, the connection between DMN network and DAN network was weakened, and the connection with VAN network was more intimate, and it was more dependent on DAN.

In the analysis of functional sub-networks involved in visual information processing, the threshold of 0.8 was utilized to determine the average connection strength with the DMN. The resulting network connections are illustrated in Figures 5A–D, which specifically focuses on the functional sub-networks associated with visual information processing. It is important to note that the connections shown in the figure do not include the connections within the DMN network itself. The figure reveals that the VAN exhibited a stronger connection with the DMN network compared to the DAN. Furthermore, within the connection to the DMN network, the dorsal-medial system demonstrated a closer association with the attention network, specifically the posterior-dorsal and medial-hemispheric regions. In the network connections observed in the group of patients, the connection between the DMN network and DAN network was weakened, while the connection with the VAN network became more prominent, indicating a greater reliance on the DAN network.

### 3.3 DTI analysis results

**Figure 6:**
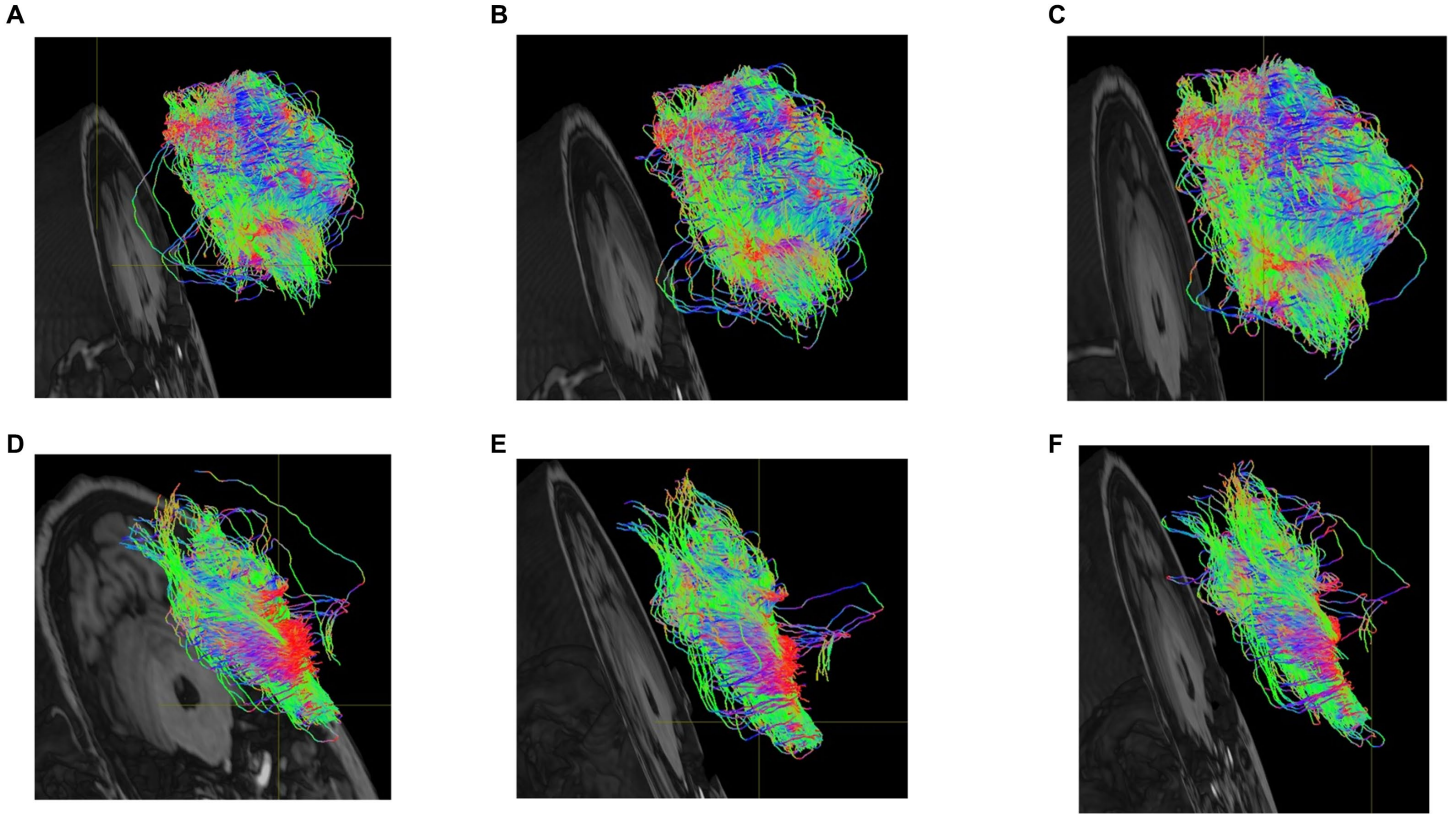
**(A)** dorsolateral superior frontal gyrus-cuneus of PD-MH group **(B)** suboccipital gyrus-insula of PD-MH group **(C)** middle temporal gyrus-amygdala of PD-MH group **(D)** dorsolateral superior frontal gyrus-cuneus of HC group. **(E)** suboccipital gyrus-insula of HC group **(F)** middle temporal gyrus-amygdala of HC group.

Fiber tracts were delineated based on the findings of brain regions exhibiting significant differences in certain functional brain networks, as presented in Table 2. The visualization figures were generated using Mrtrix 3 software on a Linux ubuntu 16.04 platform. A total of 5,000 fiber tracts were sampled for both groups. Figure 6 illustrates that, in comparison to the HC group, the PD-MH group displayed a lower density and more dispersed arrangement of fiber bundles, whereas the HC group exhibited a higher density and more compact configuration.

### 3.4 Visual reconstruction analysis results

**Figure 7:**
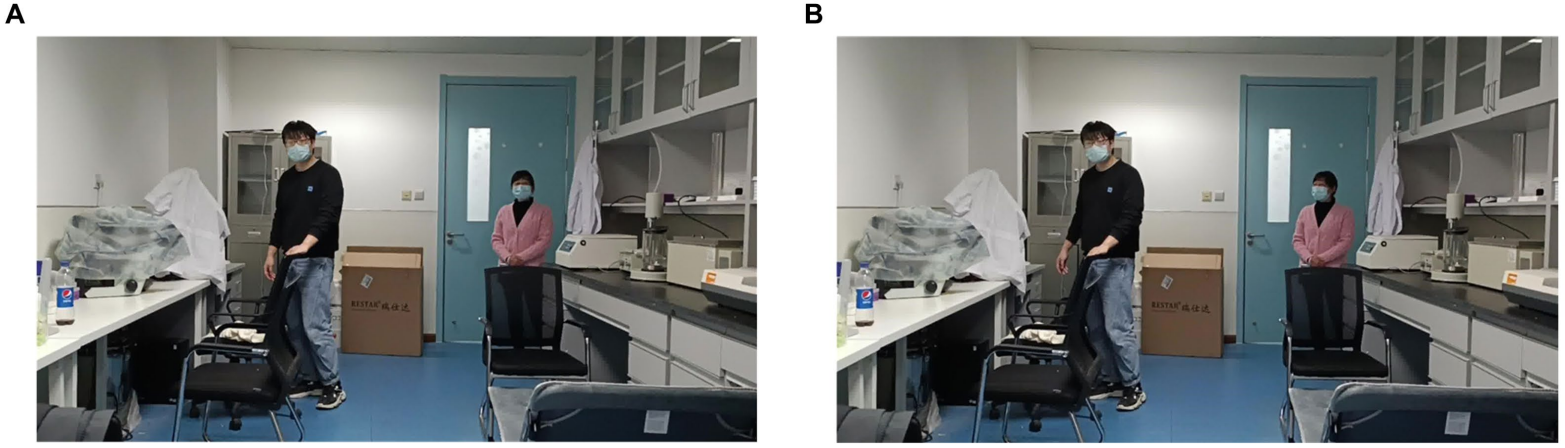
Real experimental scene of visual reconstruction. **(A)** Partner in the distance faces the PD-MH subject. **(B)** Partner in the distance does not face the PD-MH subject.

Based on the findings from the analysis of fMRI and EEG data, we identified specific channels within the DMN that are located near the dorsal subsystem. Additionally, we selected electrodes in the frontal lobe region associated with the VAN, electrodes in the occipital lobe region situated in the inferior occipital gyrus, and electrodes in the temporal lobe region linked to memory recall. These channels were utilized for the visual reconstruction of a PD-MH subject who experienced persistent hallucinations of being stared at. To minimize any potential disturbance to the subject, we opted for a slightly complex scene consisting of one person at a close distance and another person at a greater distance. Throughout the experiment, the person would either face the subject directly or have their side face visible. The actual experimental scenes are depicted in Figure 7A (subject facing) and Figure 7B (side face).

**Figure 8:**
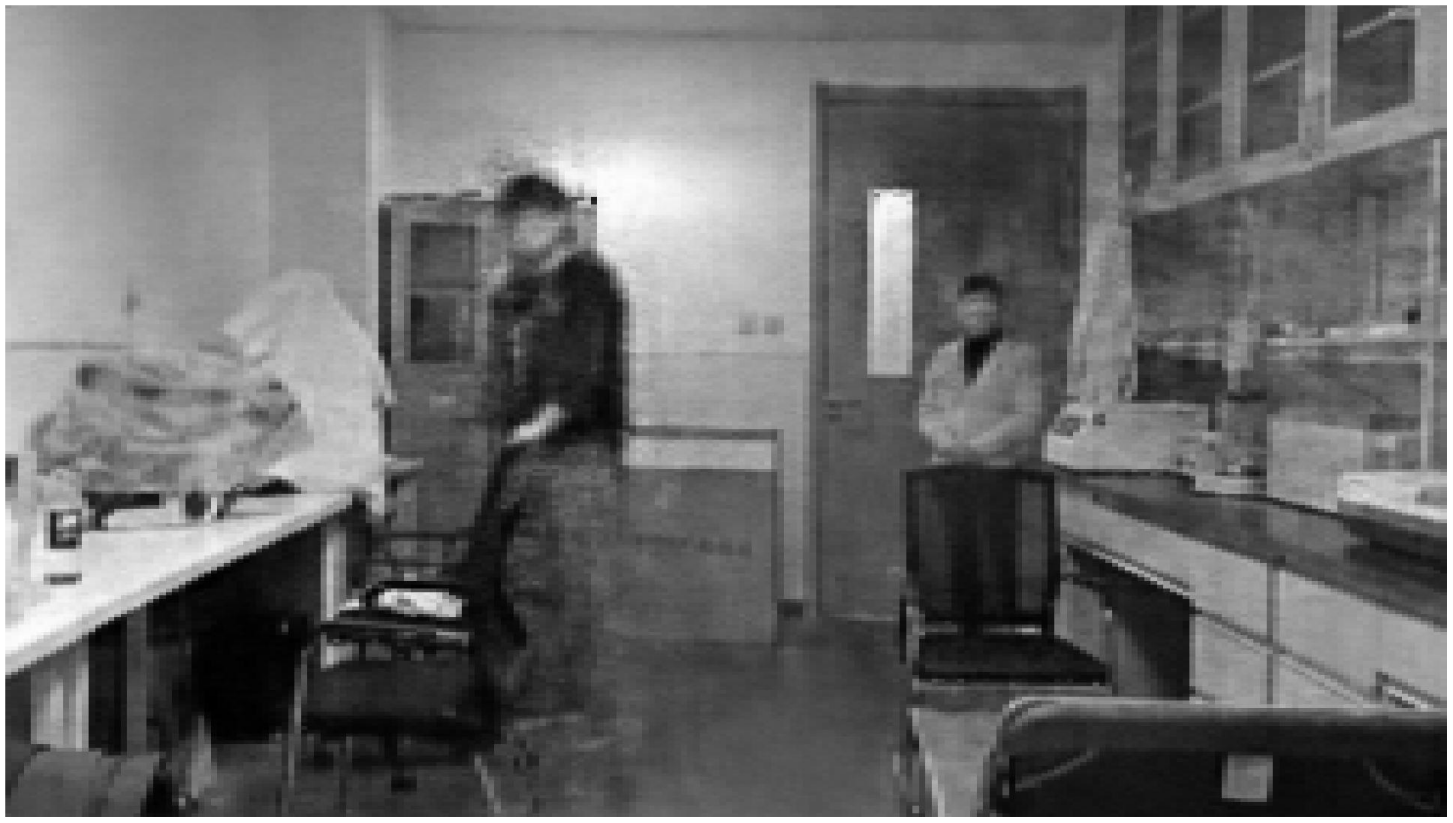
Reconstructed images of hallucinating subjects.

To classify the two scenarios (i.e., whether the partner’s face was directed toward the subject or not), we employed a deep learning network, as illustrated in Figure 8. The SSIM of Figure 8 is 0.747. However, the findings indicate that the reconstructed images consistently depict the subject facing forward and are unable to differentiate between the two scenarios.

**Figure 9:**
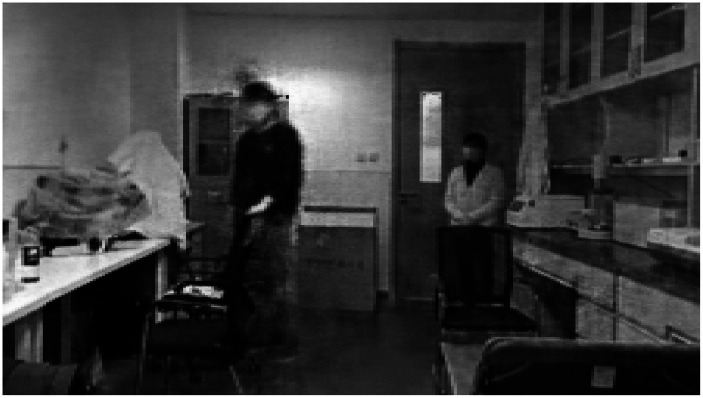
Reconstructed images of healthy subjects.

In the case of healthy subjects, when playing a previously recorded video of the same scenarios, we were able to reconstruct the two scenarios using a deep learning network. Figure 9 displays the reconstructed image of the side face scenario. Although the facial image of the distant collaborator is not highly distinct, it is evident that the subject’s face is not facing forward. The classification of the two scenarios (face or not face subject) can be achieved by utilizing EEG data from HC subjects. The SSIM of Figure 9 is 0.759, indicating that the results obtained from HC subjects are superior to those obtained from individuals with PD-MH.

## 4 Discussion

After conducting an analysis of EEG and fMRI data, significant findings were observed in the activity of different frequency bands among the three groups of subjects. Specifically, the α and θ bands exhibited enhanced activity, while the power of the Gamma γ band was weakened.

The attentional networks model proposes that CVH in PD patients are associated with impaired activation of the DAN when interpreting ambiguous percepts. This finding aligns with our own results in MH, which indicate that the DAN is weakened in individuals with MH.

The analysis of DTI data shows that MH has a more dispersed arrangement of fiber bundles in the DAN related brain regions compared to HC. The location of abnormal fiber bundles related to DAN provide a basis for choosing EEG channels containing hallucinatory information.

To establish a correlation between abnormal EEG signal and MH, we conducted an study on a PD-MH participant who experienced hallucinations of being stared at during the experiment. The EEG data from this PD-MH participant were unable to differentiate between two scenarios (presence or absence of a face) using visual reconstruction techniques. In contrast, the EEG data from a HC participant successfully distinguished between these two scenarios. This suggests that visual reconstruction techniques may offer a means of evaluating hallucinations.

Future research should explore the relationship between MH and more complex visual hallucinations. Further analysis of MH could potentially facilitate early identification of PD patients who are at risk of developing hallucinations.

## 5 Limitations

In the visual reconstruction experiment, only one subject with PD-MH was included due to the challenges associated with obtaining data on ongoing hallucinations. As a result of several participants’ inability to recall the specifics of their hallucinations, their MH characteristics are documented as unknown in Table 1. The analysis of different types of MH is limited by the small number of patients in each category.

## Data availability statement

The raw data supporting the conclusions of this article will be made available by the authors, without undue reservation.

## Ethics statement

The studies involving humans were approved by the Ethics Committee of Affiliated Brain Hospital of Nanjing Medical University. The studies were conducted in accordance with the local legislation and institutional requirements. The participants provided their written informed consent to participate in this study. Written informed consent was obtained from the individual(s) for the publication of any identifiable images or data included in this article.

## Author contributions

CL and LQ: study design, data collection, data analysis, and manuscript writing. KY: funding acquisition and supervision. WL: data collection and supervision. QL: data analysis. YC: data collection. JS and CY: manuscript editing. All authors contributed to the article and approved the submitted version.
